# Editorial: Case reports in pulmonary medicine

**DOI:** 10.3389/fmed.2023.1279945

**Published:** 2023-11-01

**Authors:** Rodrigo Torres-Castro, Suzanna Tanni

**Affiliations:** ^1^Department of Physical Therapy, Faculty of Medicine, University of Chile, Santiago, Chile; ^2^Pulmonology Division of Internal Medicine Department, Botucatu Medical School, São Paulo State University (UNESP), Botucatu, São Paulo, Brazil

**Keywords:** respiratory medicine, clinical case, pulmonary medicine, report, unique case study

Case reports are vital components of scientific literature, providing essential insights into rare or unique medical conditions, diagnostic dilemmas, and therapeutic challenges (1). There has been a growing trend in the publication of clinical case reports over the years. In the year 2000, ~42,000 case reports were published in PubMed-indexed journals. However, this number surged significantly to 89,125 by the year 2020, signifying a remarkable growth rate of close to 110%. This increase highlights the escalating importance and impact of these reports in contributing to the advancement of medical knowledge and enhancing clinical practice.

Clinical case reports correspond to articles with the lowest level of evidence on the hierarchy of various research study types (2). However, this type of publication have historically played a vital role in identifying emerging or uncommon diseases, assessing the positive and adverse outcomes of interventions, and enriching medical education (1). They provide a platform for clinicians to share unusual clusters of symptoms or presentations that might indicate the presence of a new disease or variant. The swift dissemination of such medical information can facilitate prompt public health responses, as demonstrated during outbreaks of infectious respiratory diseases like the recent COVID-19.

Medical literature is often dominated by observational studies involving larger patient cohorts, leaving rare and unusual cases underrepresented. Case reports fill this gap by presenting detailed accounts of such unique situations that can be useful for other clinicians (3). Within the domain of respiratory medicine, where unique conditions such as rare lung diseases, unconventional infections, and atypical manifestations prevail, case reports form the bedrock upon which comprehension of these puzzling illnesses is built.

The diverse range of respiratory diseases often presents diagnostic challenges due to overlapping clinical features. Case reports detailing unique diagnostic dilemmas and the strategies used to resolve them offer valuable learning opportunities for healthcare professionals (4). By sharing their experiences, clinicians can contribute to the collective knowledge, leading to improved diagnostic accuracy and timely initiation of appropriate treatment.

Case reports can provide early evidence of the effectiveness of novel therapies or management strategies for different conditions (3). In instances where patients experience suboptimal responses to common and standard treatments, alternative approaches tested in individual cases can be reported (4). While not conclusive evidence, these reports can prompt further research and eventually lead to the development of more effective treatments (4). On the other hand, these reports act as preliminary evidence, encouraging researchers to investigate potential causal links or mechanisms behind the observed outcomes. Such investigations can guide the design of more rigorous studies, eventually leading to evidence-based medical practices.

For example, in this Research Topic, we present unusual cases that can provide valuable insights for clinicians facing similar challenges in their practice. One of the cases reported highlights a refractory pneumothorax secondary to human immunodeficiency virus -associated *Pneumocystis jiroveceii* pneumonia, which exhibited a positive response to endobronchial Watanabe spigot and blood coagulation factor XIII supplementation (Koyama et al.). In the same way, others atipical respiratory infection are exposed in this Research Topic. Sun et al. described a chronic respiratory condition due to *Fusobacterium nucleatum* in pleural effusion associated with squamous cell carcinoma. Similarly, Yuan et al. presented a case with *Actinomyces graevenitzii*, Zhang et al. illustrated an abnormal *Streptococcus pneumoniae* thoracic image presentation, Peng et al. showed a hypervirulent *Klebsiella pneumonia*, and Liu and Gao described an infection with *Chlamydia psittaci* in severe pneumonia cases. All these cases had a positive clinical resolution after identification of the pathogen by molecular sequencing. Unfortunately, molecular diagnostics by next-generation sequencing is not a procedure accessible at the most respiratory centers in the world. In this context, these published cases are important to propose management of rare infection cases in respiratory field.

Another intriguing cases involved abnormalities in the thoracic anatomy. A post-COVID-19 tracheal stenosis with fibrotic bridges, leading to significant respiratory distress (Zuccatosta et al.). The absence of cartilaginous support involvement allowed for successful bronchoscopic treatment, resulting in complete and permanent resolution of the stenosis (Zuccatosta et al.). Other rare conditions are described in the biliobronchial fistula after cholecystectomy surgery case (Batalin Júnior et al.), a Kartagener síndrome with DNAH9 mutation case (Feng et al.), an anomalous systemic arterial supply to the left lower lung lobe (Wu et al.), an epithelioid hemangioendothelioma in main bronchus (Gong et al.), and tracheal lobular capillary hemangioma (Tao et al.). These reports offer important clinical knowledge and potential solutions for managing complex respiratory conditions.

Finally, this type of evidence serve as powerful teaching tools, especially in medical education (1). They provide real-world examples of clinical decision-making, the importance of thorough patient histories, and the significance of physical examinations (4). Aspiring medical professionals can learn from the experiences shared in case reports, enabling them to make more informed decisions when faced with similar clinical scenarios.

Despite representing the lowest level of evidence, clinical case reports continue to be among the most significant sources of knowledge in the biomedical field. They offer valuable insights into unusual disease presentations and the benefits of employing unconventional approaches in treatment (2). Moreover, case reports enable readers to explore novel concepts and foster innovative perspectives for their everyday clinical practice (2).

## Author contributions

RT-C: Writing – original draft, Writing – review & editing. ST: Writing – original draft, Writing – review & editing.
